# Cross-sectional studies: understanding applications, methodological issues, and valuable insights

**DOI:** 10.36416/1806-3756/e20250047

**Published:** 2025-03-18

**Authors:** Ricardo G Figueiredo, Cecilia M Patino, Juliana C Ferreira

**Affiliations:** 1. Methods in Epidemiologic, Clinical, and Operations Research - MECOR - program, American Thoracic Society/Asociación Latinoamericana del Tórax, Montevideo, Uruguay.; 2. Programa de Pós-Graduação em Saúde Coletiva, Universidade Estadual de Feira de Santana - UEFS - Feira de Santana (BA) Brasil.; 3. Department of Population and Public Health Sciences, Keck School of Medicine, University of Southern California, Los Angeles (CA) USA.; 4. Divisão de Pneumologia, Instituto do Coração, Hospital das Clínicas, Faculdade de Medicina, Universidade de São Paulo, São Paulo (SP) Brasil.

## PRACTICAL SCENARIO

Investigators from Latin America conducted a cross-sectional study to estimate the prevalence of COPD in adults ≥ 40 years of age in five major Latin American cities.^1^ Key variables, including smoking history, pre and post-bronchodilator spirometry, were measured at a single point in time in all participants. The authors of the PLATINO study reported the prevalence of COPD in São Paulo (Brazil), Santiago (Chile), Mexico City (Mexico), Montevideo (Uruguay), and Caracas (Venezuela), with prevalences ranging from 8% to 20%. 

## WHAT IS A CROSS-SECTIONAL STUDY?

A cross-sectional study is a type of observational study widely employed across diverse disciplines, and its use is particularly relevant to public health, epidemiology, and the social sciences. It provides a snapshot of a population on a single occasion and enables the analysis of associations between variables without the influence of temporal factors.^2^ This design is particularly well-suited to estimate the prevalence of diseases and health outcomes, describe population characteristics, and evaluate associations in a defined population. Since the exposure and outcome are measured concurrently and with no follow-up period, investigators analyze the distribution of outcome variables across the exposures based on biological plausibility and prior evidence.^3^

The strengths of cross-sectional studies lie in their cost-effectiveness and efficiency, as they are relatively fast to complete and inexpensive. They are also the appropriate design to estimate disease prevalence. Additionally, minimal ethical concerns arise since participants are not deliberately exposed to interventions.^2^ However, cross-sectional studies have notable limitations. Investigators cannot use them to assess disease incidence, and they are unfeasible for studying rare conditions. Importantly, cross-sectional studies do not allow investigators to evaluate causality, because it is not possible to establish whether a suspected exposure variable preceded the suspected outcome. We summarized the advantages and disadvantages of cross-sectional studies in Chart 1.

**Chart 1:** Cross-sectional studies: advantages and disadvantages

Advantages	Disadvantages
Less time-consuming; cost-effective.	Unpractical for studying rare diseases
Efficiency	Unable to assess incidence
Facilitates hypothesis generation	Pitfalls inferring causality, prognosis, or natural history of disease
Multiple outcomes and exposures can be studied simultaneously	Selection bias risk
Minor ethical concerns	Cannot establish temporal relationships between exposure and outcome
Suitable for large sample sizes	Potential confounding factors may bias associations

## RESEARCH QUESTIONS SUITABLE FOR CROSS-SECTIONAL STUDIES

Selecting the appropriate study design for a specific research question is a critical and often challenging step in developing the research plan. Cross-sectional studies help estimate the prevalence of a condition or outcome within the study population. This design is well-suited for descriptive and exploratory analyses. Large-scale epidemiological studies have leveraged this approach to provide valuable insights into the distribution of risk factors and the impact of social determinants of health on the development of prevalent diseases, contributing to the formulation of public health policies worldwide. Diagnostic test accuracy studies are particularly appropriate for a cross-sectional design.

Although cross-sectional studies are not suitable for research questions that evaluate causality, they can be employed to investigate associations among variables. In this context, the decision to label variables as exposures or outcomes is guided by the investigator’s cause-and-effect hypothesis rather than determined by the study design itself.^3^

## INTERPRETATION OF FREQUENCY AND ASSOCIATION MEASURES IN CROSS-SECTIONAL STUDIES

In contrast to longitudinal studies, cross-sectional studies are designed to capture prevalence, representing the proportion of individuals with a disease or condition at a specific point in time among a population of interest. Investigators should report the number of events in participants with and without the exposure and provide prevalence precision with a 95% confidence interval (95% CI).^4^ The prevalence of an outcome can be compared between exposed and unexposed, providing measures of association such as the odds ratio (OR) and the prevalence ratio (PR). In the practical scenario described, the authors reported that the prevalence of COPD ranged from 7.8% (95% CI: 5.9-9.7) in Mexico City to 19.7% (95% CI: 17.2-22.2) in Montevideo.

Drawing inferences regarding causality, prognosis, or natural history of disease from cross-sectional data requires caution. A variable associated with the outcome of interest may be a causal factor, but it could also simply reflect an association with the disease’s duration.^3^ Exposures may be influenced by confounding factors that also impact the outcome. Therefore, it is crucial to identify potential confounders during the study design phase and apply appropriate statistical methods to minimize distortion in the associations between the variables of interest.

## KEY MESSAGES

1. Cross-sectional studies provide valuable insights into prevalence and associations within populations.
2. Although these studies provide a population snapshot at a specific point, their design limits inferences regarding causality.
3. Quality control during the conception and conduct of cross-sectional studies and awareness of its limitations are crucial to maximizing their utility in advancing evidence-based practice.
